# Translational reprogramming in tumour cells can generate oncoselectivity in viral therapies

**DOI:** 10.1038/ncomms14833

**Published:** 2017-03-16

**Authors:** Eneko Villanueva, Pilar Navarro, Maria Rovira-Rigau, Annarita Sibilio, Raúl Méndez, Cristina Fillat

**Affiliations:** 1 Gene Therapy and Cancer, Institut d'Investigacions Biomèdiques August Pi i Sunyer (IDIBAPS), Rosselló 149-153, 08036 Barcelona, Spain; 2 Cancer Research Program, IMIM (Hospital del Mar Medical Research Institute), Dr Aiguader 88, 08003 Barcelona, Spain; 3 Centro de Investigación Biomédica en Red de Enfermedades Raras (CIBERER), 08036 Barcelona, Spain; 4 Institute for Research in Biomedicine (IRB Barcelona), The Barcelona Institute of Science and Technology, Baldiri Reixac, 10, 08028 Barcelona, Spain; 5 Institució Catalana de Recerca i Estudis Avançats (ICREA), 08010 Barcelona, Spain

## Abstract

Systemic treatment of cancer requires tumour-selective therapies that eliminate cancer cells yet preserve healthy tissues from undesired damage. Tumoral transformation is associated with profound effects in translational reprogramming of gene expression, such that tumour-specific translational regulation presents an attractive possibility for generating oncoselective therapies. We recently discovered that mRNA translational control by cytoplasmic polyadenylation element-binding proteins (CPEBs) is reactivated in cancer. Here we present a novel approach to restrict genetic-engineered therapies to malignant tissues based on CPEB translational regulation of target mRNAs. We demonstrate that tumour reprogramming of CPEB-mediated mRNA stability and translational regulation modulates tumour-specific expression of viral proteins. For oncolytic adenoviruses, insertion of CPE regulatory sequences in the 3′-untranslated region of the *E1A* gene provides oncoselectivity, with full potency in cancer cells but attenuated in normal tissues. Our results demonstrate the potential of this strategy to improve oncolytic virus design and provide a framework for exploiting CPE-regulated transgenes for therapy.

## Abstract (web summary)

mRNA translational control by CPEBs is reactivated in cancer. Here, the authors show that the insertion of CPE regulatory sequences in the 3′ UTR of the E1A viral gene induces oncoselectivity, with oncolytic potency in cancer cells but attenuated toxicity in normal tissues.

Regulation of transgene and viral protein expression is required to increase the safety and efficacy of gene and viral therapies. Delivery and expression of transgenes with anticancer activity, or the use of conditionally replicating viruses for cancer therapy, must be specific for tumours to avoid side effects to healthy tissues. Most efforts to achieve such selective control have been based on the use of tumour-specific promoters^1^ and, more recently, by the engineering of target site-recognizing, tissue-specific microRNA (miRNA)^2^,^3^,^4^,^5^,^6^. Although both strategies contribute highly to tumour selectivity, it has become evident that the post-transcriptional regulation of specific mRNA subpopulations contributes substantially to the broad expression changes of genes responsible for the cancer phenotype^7^. Thus, the translational reprogramming of tumour cells has been proposed as a potential target for tumour-specific drugs^8^. These tumour-specific translational profiles could therefore be used to generate tumour specificity to transgene and viral protein expression.

One of the mechanisms to regulate the translation of specific subpopulations of mRNAs is through the presence of *cis*-acting elements in the 3′-untranslated region (3′-UTRs) of mRNAs, such as the cytoplasmic polyadenylation element (CPE; 5′-UUUUA_1–2_U-3′). The CPE is bound by CPE-binding proteins (CPEBs), a family of four members conserved in their RNA-recognition domain but distinct in their regulatory motifs (reviewed in refs 9, 10). All four CPEBs recognize RNA via tandem RNA recognition motifs located in their C-terminal halves that are followed by ZZ domains^11^. The RNA recognition motifs define two CPEB subfamilies (CPEB1 and CPEB2–4). Both CPEB subfamilies regulate overlapping populations of mRNAs and recognize similar *cis*-acting elements, albeit with different affinities^12^,^13^,^14^,^15^. The N-terminal domain is highly variable in both length and composition across various CPEB orthologues and paralogues^16^ and contains all the identified regulatory motifs, including phosphorylations^17^,^18^, monoubiquitination^15^ and sumoylation^19^. These post-translational modifications dictate whether CPEBs act as translational repressors or promote cytoplasmic polyadenylation and subsequent translational activation (reviewed in refs 9, 10). In turn, the specific arrangement of CPEs, in number and in their relative position to other CPEs and the polyadenylation signal^20^, together with the presence of additional *cis*-acting elements such as AU-rich elements (AREs)^21^, quantitatively determine whether a particular mRNA is repressed or activated in a combinatorial code that responds to the activation of specific CPEBs^12^. Although CPEBs have been mainly characterized as regulators of maternal mRNAs during the transcriptionally silent germ cells, they also regulate cytoplasmic polyadenylation in somatic and tumoral cells^13^,^22^,^23^,^24^,^25^. In fact, CPEBs are extremely conserved within vertebrates (96% for CPEB1 and 99% for CPEB4), the CPEs and the CPE-combinatorial code are conserved and functionally replaceable from frogs to mammals and promote translational repression and activation in rodents and humans^13^,^21^,^26^,^27^. CPEB levels and activities are differentially regulated in tumours^9^,^28^. CPEB1 levels are decreased in several human cancers^29^,^30^, and reduced levels of this protein have been associated with increased malignancy both in human^29^ and mouse^23^. In contrast, high CPEB4 expression levels in pancreatic ductal adenocarcinoma and glioblastoma tumours correlated with stimulation of tumour growth, angiogenesis and decreased survival^14^. Furthermore, CPEB4 has been identified as one of the seven genes with an expression profile that is associated with colorectal cancer^31^.

Here we show the feasibility of using the tumoral reprogramming of CPE-mediated translational regulation to provide tumour selectivity to transgene expression. We have engineered a particular CPE arrangement that activates translation in tumour cells while promoting translational repression in non-transformed cells. We then generated a modified adenovirus in which the E1A protein expression is regulated by CPEBs, to obtain oncoselectivity and attenuated toxicity in non-transformed tissues. This novel targeting modality increases the therapeutic index of an oncolytic adenovirus and provides a new paradigm for its applicability to gene transfer-based therapeutic approaches.

## Results

### CPEs provide oncoselectivity to replicative adenoviruses

**Figure 1:**
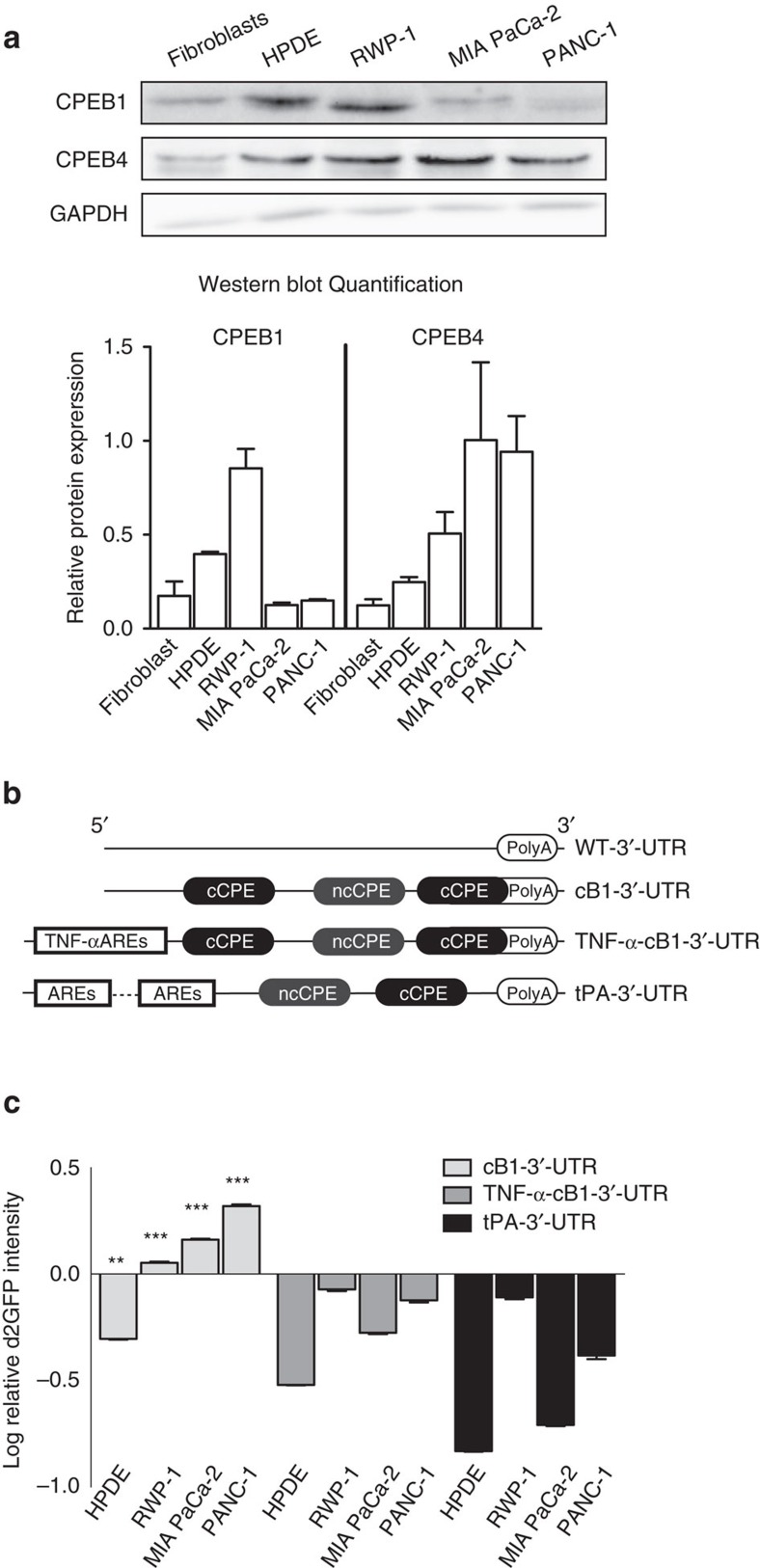
CPEs containing 3′-UTR confer *in vitro* oncoselectivity to engineered transgenes. (**a**) The upper panel shows representative western blots showing CPEB1 and CPEB4 expression in pancreatic primary fibroblasts, normal cells (HPDE) and tumour cells (RWP-1, MIA PaCa-2 and PANC-1). The lower panel shows quantification of CPEB1 and CPEB4 signals normalized to glyceraldehyde 3-phosphate dehydrogenase (GAPDH). (**b**) Schematic representation of the assessed 3′-UTR. Regulatory sequences are indicated. (**c**) Quantification of relative d2EGFP/dRFP fluorescence intensity levels in cell lines transduced with the indicated lentiviruses and relative to the mean intensity/content of the d2EGFP/dRFP from cells transduced with Lv-WT 3′-UTR. Data are shown as mean±s.e.m. from three independent biological replicates and were analysed by a linear mixed model fit by REML and a Tukey's contrast test to assess the significance of the differences. **P<0.01, ***P<0.001. cCPE, consensus CPE; ncPCE, nonconsensus CPE.

To identify the optimal sequences required for CPEB-dependent tumour selectivity, we tested chimeric mRNAs with three combinations of 3′-UTRs fused to the d2EGFP open reading frame. These mRNAs were expressed in a battery of normal (HPDE) and tumour (RWP-1, PANC-1 and MIA PaCa-2) pancreatic cells expressing variable levels of CPEB1 and CPEB4 (Fig. 1a). The first UTR was derived from *Xenopus* cyclin B1 (cB1) 3′-UTR mRNA and contained two consensus CPEs and one nonconsensus CPE. This CPE arrangement promotes both translational repression by unphosphorylated CPEB1 and translational activation by CPEB4^12^,^13^,^20^. The second UTR was synthetized by combining cB1 CPEs with an ARE sequence that opposes CPE-mediated polyadenylation and translational activation from the tumour-necrosis factor-α (TNF-α) 3′-UTR mRNA (TNF-α-cB1). The third UTR was generated from a fragment of the tissue plasminogen activator (tPA) 3′-UTR mRNA that contains two CPEs and two ARE sequences^14^. (Fig. 1b and Supplementary Table 1).

Lentiviruses expressing the d2EGFP with the indicated 3′-UTRs or a control wild-type (WT)-3′-UTR (without CPEs) and a lentivirus with a destabilized dRFP bearing a control 3′-UTR (WT) were used to coinfect the non-tumour HPDE cells and the pancreatic cancer cell lines (Supplementary Fig. 1a). For each combination of UTR and cell line, the resulting d2EGFP-to-dRFP expression ratio indicated that cB1 was the only 3′-UTR promoting a differential translational control in normal cells compared to cancer cells. Thus, cB1-3′-UTR repressed the expression of d2EGFP in HPDE cells as compared to the dRFP control 3′-UTR, whereas it promoted activation in the cancer cells, with a stronger effect in PANC-1 (Fig. 1c). Interestingly, for the four cell lines, the d2EGFP/dRFP ratio was proportional to the CPEB4/CPEB1 ratio (Fig. 1a). As expected from the role that the poly(A) tail has on mRNA stability, reduced mRNA levels of d2EGFP-cB1-3′-UTR were detected in HPDE cells as compared to tumour cells (Supplementary Fig. 1b). On the other hand, the two 3′-UTRs with ARE sequences led to reduced d2EGFP expression in all the cell lines, indicating that the destabilization effect of the ARE dominates over the effects of CPE regulation (Fig. 1c).

**Figure 2:**
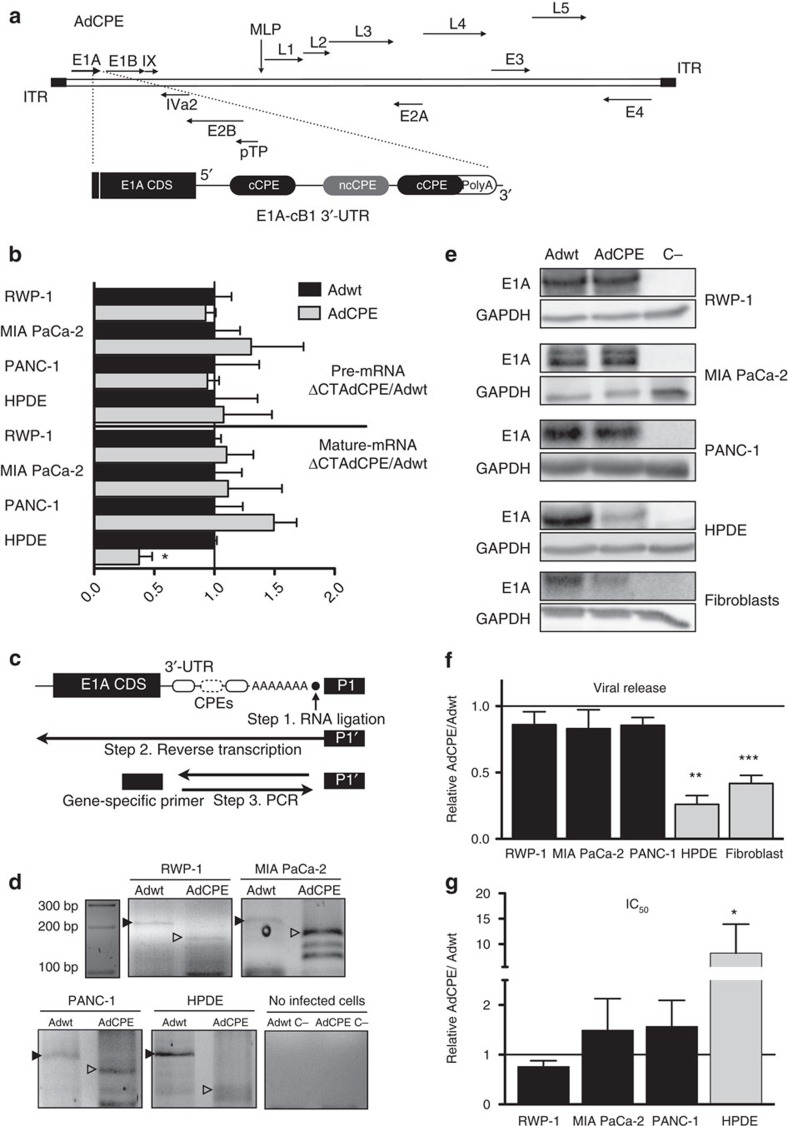
CPEs in the 3′-UTR of E1A confers *in vitro* oncoselectivity to adenoviruses. (**a**) Schematic representation of the whole adenoviral genome with the CPE-engineered sites in the *E1A* viral gene. (**b**) Quantification of E1A pre-mRNA and mature mRNA 4 h postinfection in the indicated cell lines. qPCR data are shown as the mean±s.e.m. of five independent biological replicates. *P<0.05 (two-tailed Mann–Whitney test). (**c**) Schematic representation of the RNA ligation-coupled RT–PCR technique used to assess poly(A) tail lengths. (**d**) Polyadenylation of E1A mRNA 4 h postinfection in tumour RWP-1, PANC-1 and MIA PaCa-2 cells or in non-tumour HPDE. Retarded migration indicates longer polyadenylation. Adwt C− and AdCPE C− correspond to an RNA mix from non-infected cells amplified with specific primers for Adwt and AdCPE, respectively. (**e**) Representative E1A western blots of pancreatic tumour cells (RWP-1, MIA PaCa-2 and PANC-1) and non-tumour cells (HPDE and fibroblasts) infected with Adwt and AdCPE at 72 h postinfection. (**f**) Quantification of viral production in supernatant 72 h postinfection in tumour (RWP-1, MIA PaCa-2 and PANC-1) and non-tumour cells (HPDE and pancreatic fibroblasts) infected with Adwt and AdCPE 72 h postinfection. qPCR data are shown as mean±s.e.m. of five independent biological replicates. **P<0.01 and ***P<0.001 (two-tailed Mann–Whitney test). (**g**) Cytotoxicity assay in the indicated cell lines. Half-maximal inhibitory concentration (IC_50_) was calculated for each cell line from dose–response curves. Data are shown as mean±s.e.m. from five independent biological replicates. *P<0.05 (two-tailed Mann–Whitney test).

Next, we took advantage of the specific regulation promoted by the cB1-3′-UTR to generate an oncoselective replication-competent adenovirus Ad5. For this, we targeted viral replication by designing a virus in which the translation of E1A mRNA was regulated by CPEBs. The adenoviral immediate-early *E1A* gene was selected because it is the first gene transcribed after an adenoviral infection and thus acts as a master transcriptional regulator of further early viral genes and modifies several cell host functions required for viral DNA replication. We then replaced the WT-3′-UTR of the viral E1A coding sequence with the cB1-3′-UTR to give us AdCPE (Fig. 2a). Substitution of *E1A* WT-3′-UTR by cB1-3′-UTR had no effect on the transcription of this gene, as shown by the equal levels of pre-mRNA for both 3′-UTRs in normal and cancer cell lines (Fig. 2b). However, when the steady-state levels of mature transcripts were compared, we found E1A-cB1-3′-UTR mRNA to be significantly lower in HPDE cells as compared to E1A-WT-3′-UTR mRNA as well as reduced with respect to E1A- cB1-3′-UTR mRNA in tumour cells (Fig. 2b). This suggests a specific destabilization of the mRNA-containing CPEs in the non-tumour cells. Because the destabilization of the CPE-containing c-myc mRNA in non-transformed cells has been directly associated with its cytoplasmic deadenylation^25^, we measured the polyA tail length of the different E1A transcripts in the four cell lines by RNA ligation-coupled PCR with reverse transcription (RT–PCR) analysis (Fig. 2c). We found that the CPE-mediated destabilization of the E1A-cB1 mRNA in HPDE cells was associated with a shorter polyA tail (Fig. 2d), which resulted in reduced E1A protein expression from the cB1-3′-UTR mRNA as compared with the WT-3′-UTR (Fig. 2e). Accordingly, AdCPE-infected non-transformed cells (HPDE) showed a strong reduction in viral genome copy numbers (Fig. 2f) and decreased cytotoxicity (increased half-maximal inhibitory concentration (IC_50_); Fig. 2g) as compared to the control virus Adwt. The lowest performance of AdCPE was also observed in human primary fibroblasts and the non-tumoral human kidney epithelial HK-2 cells (Fig. 2e,f and Supplementary Fig. 2). However, in the tumour cells, cB1-3′-UTR was as efficient as the WT-3′-UTR in supporting optimum levels of viral-driven E1A expression (Fig. 2e), which in turn resulted in equal viral genome copy numbers and IC_50_ from both 3′-UTRs (Fig. 2f,g). CPEB-mediated control of E1A expression and viral release was also observed in AdCPE-infected colorectal and glioblastoma cells as well as in the patient-derived pancreatic tumour CP15 (Supplementary Fig. 2). The similar effects of Adwt and AdCPE in cancer cells also highlight that the small size of the cB1-3′-UTR did not compromise virus fitness and packaging efficiency.

### CPE-mediated oncoselectivity is provided by CPEB4

**Figure 3:**
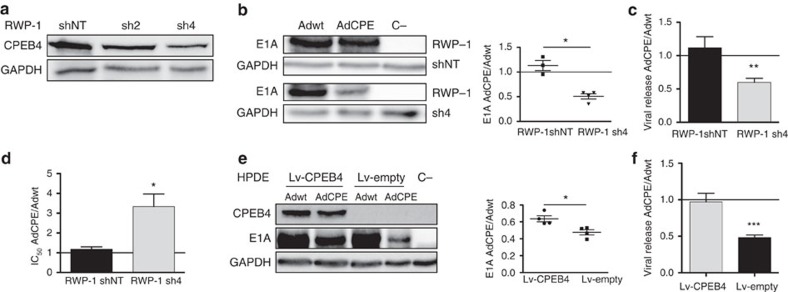
CPEB4 regulates AdCPE E1A expression and viral fitness. (**a**) Representative western blot of CPEB4 protein downregulation in RWP-1 tumour cells. shNT, non-targeted; sh2 and sh4, two different sh sequences against CPEB-4. (**b**) Representative western blot of E1A expression in RWP-1 shNT and RWP-1 sh4, infected with Adwt and AdCPE 72 h postinfection. Quantification of the E1A signal was normalized to glyceraldehyde 3-phosphate dehydrogenase (GAPDH) and expressed as relative values of AdCPE/Adwt (*n*=4). *P<0.05. (**c**) Quantification of relative viral production (AdCPE/Adwt) in the supernatant of sh4 and shNT RWP-1-infected cells 72 h postinfection. Data are shown as mean±s.e.m. from five independent biological replicates. **P<0.01 (two-tailed Mann–Whitney test). (**d**) Cytotoxicity assay in sh4 and shNT RWP-1 cells infected with Adwt or AdCPE for 72 h. IC_50_ was calculated for each cell line from dose–response curves. Data are shown as mean±s.e.m. from six independent biological replicates. *P<0.05 (two-tailed Mann–Whitney test). (**e**) The left panel shows a representative western blot of the E1A protein in non-tumour HPDE cells transduced with a lentivirus-expressing CPEB4 (Lv-CPEB4) or a control lentivirus (Lv-empty) and infected with Adwt or AdCPE. E1A content was evaluated 72 h postinfection. The right panel shows quantification of the E1A signal that was normalized to GAPDH and expressed as relative values of AdCPE/Adwt (*n*=5). *P<0.05. (**f**) Quantification of relative viral genome release in HPDE supernatants from cells transduced with Lv-CPEB4 or Lv-empty and infected with Ad-WT or AdCPE for 72 h. Data are shown as mean±s.e.m. of five independent biological replicates. ***P<0.001 (two-tailed Mann–Whitney test).

To determine whether the higher CPEB4 levels in tumour cells caused the specificity of cB1-3′-UTR regulation of mRNA translation, we first confirmed the specificity of CPEB4 binding to the CPEs in cB1-3′-UTR. As shown by RIP, CPEB4 bound cB1-3′-UTR WT in a CPE-dependent manner. In contrast, HuR, an ARE-binding protein that promotes mRNA stabilization, displayed almost negligible binding to cB1-3′-UTR WT that was not affected by the mutations that inactivate the CPE (Supplementary Fig. 3). Then, we knocked down CPEB4 in tumoral RWP-1 cells (RWP-1-sh4) and compared it with a control non-target shRNA (RWP-1-shNT) (Fig. 3a). Depletion of CPEB4 (RWP-1-sh4) caused a significant reduction of E1A protein levels expressed from AdCPE (Fig. 3b), with the subsequent reduction of viral replication (Fig. 3c) and increased IC_50_ values (Fig. 3d). On the other hand, Adwt was unaffected by a CPEB4 knockdown (Fig. 3b–d). Similar results were observed in HCT-116 colorectal and T98 glioblastoma cells (Supplementary Fig. 4). A reverse approach, of overexpressing CPEB4 in non-tumour cells (HPDE), partially rescued the expression of E1A from AdCPE (Fig. 3e) and viral replication (Fig. 3f), as compared to Adwt. Therefore, high levels of CPEB4 seem to be required for the oncoselective behaviour of the engineered virus and to prevent the CPE-mediated translational repression observed in non-tumour cells.

### Oncoselectivity of AdCPE *in vivo*

**Figure 4:**
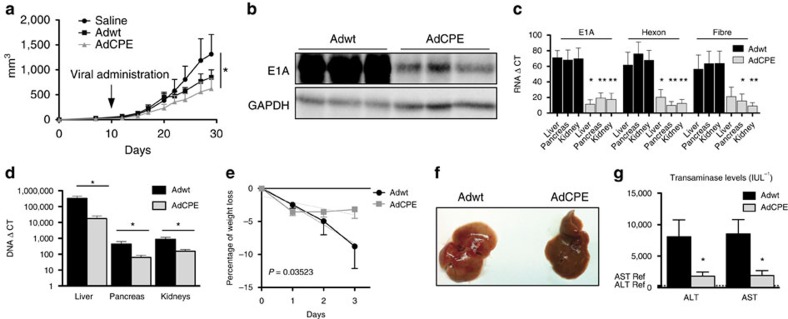
AdCPE replication is attenuated in mouse tissue, displays reduced toxicity and triggers strong antitumour activity. (**a**) Follow-up of tumour volumes in mice bearing RWP-1 xenografts intravenously treated with saline (*n*=8) or with a single injection of 2 × 10^10^ vp per mouse Adwt (*n*=8) or of 2 × 10^10^ vp per mouse AdCPE (*n*=8). (**b**–**g**) Adwt and AdCPE were intravenously delivered to WT C57BL/6 mice at 2 × 10^10^ vp per mouse (*n*=6 per group). Three days later, animals were killed and organs were isolated. (**b**) Representative E1A western blot from livers of infected mice. (**c**) Quantification of early (E1A) and late (hexon and fibre) viral mRNA content in the liver, pancreas and kidneys of infected mice by qPCR (*n*=6). *P<0.05; **P<0.01. (**d**) Viral DNA quantification by qPCR in the indicated mice tissues. Data are shown as mean±s.e.m. (*n*=6). *P<0.05. (**e**) Body weight variation of infected mice. Differences between slopes were significant (*P*=0.036). (**f**) Representative macroscopic images of livers from infected mice. (**g**) Serum transaminases AST and ALT levels (*n*=6). Normal aspartate aminotransferase (AST) and alanine aminotransferase (ALT) levels in C57BL/6 mice are indicated in dashed lines and correspond to 313.5 and 69.5 IU L^−1^, respectively.

To investigate whether AdCPE provides oncolytic specificity *in vivo*, nude mice carrying subcutaneous tumours were intravenously injected with Adwt or AdCPE viral particles, and tumour growth was monitored. In consonance with the results obtained in cell culture models, we found that AdCPE produced a significant inhibition of tumour growth that was similar or greater than that of Adwt (Fig. 4a and Supplementary Fig. 5). Interestingly, at high viral doses at which Adwt compromises mice survival^32^, the antitumour effect of AdCPE was very strong (Supplementary Fig. 6). While the efficiency of an equal dose of AdCPE and Adwt in targeting tumour cells was similar or even increased in AdCPE, the *in vitro* results with HPDE cells indicate that AdCPE should have a much reduced adenovirus-associated toxicity. To test the AdCPE effects on healthy tissues *in vivo*, we intravenously injected immunocompetent mice with Adwt and AdCPE, at the same viral dose used in the xenograft experiments. Three days later, we quantified viral proteins and viral genomes in the liver, pancreas and kidney. E1A protein levels were significantly reduced in the liver of AdCPE-treated mice, as compared to Adwt (Fig. 4b). Quantification of mRNA of E1A, hexon and fibre in the liver, pancreas and kidney revealed significantly reduced levels of the three mRNAs in AdCPE-injected mice as compared to Adwt (Fig. 4c). Consistent with *in vitro* data, E1A pre-mRNA levels in liver extracts from AdCPE and Adwt were similar, whereas the mature mRNA was reduced in AdCPE mice (Supplementary Fig. 7a). These results indicate that the CPE-dependent destabilization of viral mRNA is recapitulated *in vivo*. Despite the impairment of human Ad5 to productively replicate in mice, a low level of replication has been proposed to occur^33^, which is sufficient to detect differences between AdCPE and Adwt. Analysing viral genomes in the liver, pancreas and kidney showed, as expected, that most of the virus was retained in the liver, but that viral genomes were also present in the other organs. Interestingly, animals injected with AdCPE had a reduced viral content (of 18-fold in the liver, 7-fold in the pancreas and 6-fold in the kidney) as compared to Adwt (Fig. 4d). This effect was not associated with differences in viral titres, since the number of viral genomes that reached the liver at 4 h after injection, previous to any replication event, was similar between AdCPE- and Adwt-injected mice (Supplementary Fig. 7b). The attenuated activity of AdCPE in murine healthy tissues was further confirmed in human primary hepatocytes, a model fully permissive for adenoviral replication (Supplementary Fig. 8). Thus, AdCPE activity is highly impaired in normal tissues when compared to Adwt.

Finally, to study liver damage-associated toxicity, which is one of the major side effects of Ad-expressing E1A, we analysed body weight, macroscopic liver appearance and serum parameters in mice after intravenous delivery of Adwt or AdCPE. Adwt caused a progressive loss of weight, whereas AdCPE led to a decrease in body weight by day 1, which then remained stable for the following days (Fig. 4e). Livers injected with Adwt showed a steatotic appearance that was not observed in AdCPE livers (Fig. 4f). Aspartate aminotransferase and alanine aminotransferase enzyme activities showed a remarkable increase in Adwt-injected animals with respect to the saline group, whereas a 4-fold lower induction was detected in AdCPE-injected mice (Fig. 4g). Altogether, these results indicate a reduced toxic profile of the AdCPE virus as compared to Adwt, while maintaining its oncolytic potential.

## Discussion

Oncolytic viruses are advancing to clinical trials and are being envisioned as breakthrough agents in oncology in the near future. Accordingly, optimized engineered viruses are under development to maximize their anticancer effects^34^. However, as their potency increases, so do potentially associated toxicities, pointing to the need to develop highly tumour-specific viruses. We show that engineering adenoviruses with CPE regulatory elements, to control E1A expression post-transcriptionally, resulted in attenuated viral activity in normal cells while maintaining, or even increasing, potency in cancer cells. The specificity of the antitumour response is directly derived from the ectopic expression of CPEB4 in tumours^14^. Thus, depletion of CPEB4 attenuates viral activity in tumour cells, while its overexpression in non-transformed cells increases viral replication. In turn, the levels of CPE-mediated expression may be further increased by reduced levels of CPEB1. These results are consistent with a scenario in which CPEB1 in non-transformed cells (in which its levels are high) represses CPE-containing mRNAs^22^. In tumour cells, on the other hand, CPEB1 levels are reduced^9^,^28^, while CPEB4 levels are increased^14^, which promotes cytoplasmic polyadenylation and thereby increases mRNA stability and translation of CPE-regulated transcripts. Importantly, viral genome replication is dependent on CPEB4 activity and can only occur in tumour cells. This leads to the production of fully competent viruses in tumour cells but attenuated ones in healthy tissues. Accordingly, the CPE-regulated virus maintains its oncolytic capacity in tumour cells while significantly reducing its damage to non-tumour tissues, which is mainly in the liver as this is the target organ of adenovirus sequestration on intravascular delivery. This increased selectivity allows for an enhanced therapeutic index, since increased antitumour capacity was obtained after AdCPE treatment at viral doses that, with Adwt injection, compromised mice survival.

We demonstrate here a novel approach to fine-tuning protein expression in a tumour-selective manner by exploiting the post-transcriptional reprogramming of gene expression in tumour cells to control selectivity of an oncolytic virus. Our results have been validated in pancreatic cancer models. However, since CPEB4 is overexpressed in several tumours, such as gliomas and colorectal cancers^14^,^31^, this oncoselective strategy may be valid for many solid tumours. This approach may overcome some of the limitations associated with other commonly used postentry viral targeting approaches. Taking advantage of a tumour-selective control by tumour-specific promoters is confronted with the fact that most of the genome is constantly transcribed at low levels, and is a strategy restricted to viruses that rely on the cellular transcription machinery but not for viruses that use virally encoded polymerases for replication in the cytoplasm, such as the measles virus and vaccinia virus^34^. The alternative postentry strategy to regulate viral replication is a negative targeting approach based on the miRNA expression in normal tissues to restrict viral replication of miRNA-target site-engineered viruses. Indeed, this is a very versatile approach for many different viruses and is highly efficient. However, the evolution of escape mutants in miRNA-targeted viruses, or the potential off-target effects on the host miRNA machinery, have been proposed as potential caveats^6^. Interestingly, no mutations were found in the CPE-regulated adenovirus under evolutionary pressure (Supplementary Fig. 9). To achieve a tight restriction of replication to tumour cells, it should be possible to combine different targeting strategies such as regulation by more than one viral gene^3^ or the introduction of transcriptional and post-transcriptional control in the same viral gene^35^. In this line, a double E1A-engineered virus, with the uPAR promoter and the CPE post-transcriptional control, displayed additivity in a non-tumoral context (Supplementary Fig. 10).

This novel tropism-modified strategy presented here provides a framework for exploiting CPE-regulated transgenes to achieve cancer cell-specific expression in therapeutic gene-transfer-based strategies, or to attenuate viruses for vaccine purposes. This technology could also extend to other viruses of interest in virotherapy that require attenuation in particular tissues, such as reoviruses in heart^36^ or coxsackievirus A21 in the muscle^5^.

Altogether, our data provide a novel paradigm for the development of tumour-specific viruses and provide the proof of principle that CPEB-dependent regulation can be exploited to attenuate viral toxicity, by preventing the spread of the virus in normal tissues, without perturbation of the antitumour efficacy.

## Methods

### Cells lines

Pancreatic cell lines PANC-1 and MIA PaCa-2, glioblastoma cell lines T98 and U87, colorectal carcinoma cells HCT-116 and DLD-1 and embryonic kidney cell lines HEK293, 293T and RPE (retinal pigment epithelial) cells were obtained from the American Type Culture Collection (ATCC, Manassas, VA, USA). RWP-1 and CP15 cells were derived from human pancreatic adenocarcinoma biopsies perpetuated as xenograft in nude mice^37^,^38^. Non-tumour fibroblasts were kindly provided by Dr Eva Vaquero (Institut d'investigacions Biomèdiques August Pi i Sunyer, Barcelona, Spain). RWP-1, CP15, PANC-1, MIA PaCa-2, T98, U87, fibroblasts and RPE cells were maintained in DMEM supplemented with 10% fetal bovine serum (Gibco-BRL, Carlsbad, CA, USA). DLD-1 and HCT-116 cells were maintained in RPMI-1640 (Gibco-BRL) or McCoy's 5A (Gibco-BRL) medium, respectively, supplemented with 10% fetal bovine serum. Immortal human pancreatic duct epithelial HPDE cells, kindly provided by Dr F.X. Real (CNIO, Madrid, Spain), were cultured and maintained as reported^39^. Human hepatocytes were obtained from Biopredic International (St Gregoire, France) and maintained according to the manufacturer's instructions. RWP-1 shNT, sh2 and sh4 were described previously^14^. HCT-116 and T98 shNT and sh4 cells were generated by transducing parental cells with shNT- and sh4-expressing lentiviruses^14^. HPDE-CPEB4-expressing cells were established by transducing parental cells with CPEB4 recombinant lentivirus. Mycoplasma contamination was tested by PCR routinely.

### Lentiviral constructs with a modified 3′-UTR

The polyadenylation sequences (4,193–4,199 and 4,423–4,429) from the pLS-CG lentiviral vector (Addgene; no. 12161) were mutagenized with the help of the QuickChange II Site-Directed Mutagenesis Kit (Stratagene, Wilmington, NC, USA) according to the manufacturer's instructions (see Supplementary Table 1 for primers). Mutagenized sequences were verified by DNA sequencing.

*d2EGFP* and *d2RFP* genes were cloned by PCR with specific primers incorporating the *Age*I and *Xho*I restriction sites. The 3′-UTR of tPA was PCR amplified using specific primers with sequences for the *Xho*I restriction enzyme at both ends. The 3′-UTR of cB1 was designed in the reverse primer of *d2EGFP* gene with a sequence for the *Xho*I restriction enzyme. The 3′-UTR with the TNF-α ARE sequences was generated by amplifying the previously generated cB1 3′-UTR with a primer containing the TNF-α main ARE sequence upstream of the CPE sites.

All primer sequences are listed in Supplementary Table 1. The indicated sequences were cloned into the pLS-CG lentiviral vector. The newly generated vectors were sequenced to confirm the DNA sequence.

### Lentiviral production

Lentiviral particles were generated by cotransfection of plasmids pCMVAR8.91, pVSV-G and the pLS-CG-derived constructs, or a lenitiviral plasmid expressing CPEB4, in 293T cells by the calcium/phosphate DNA precipitation method (Clontech, Mountain View, CA, USA). Supernatants from 293T-transfected cells were collected at 24 and 48 h, filtered and processed for purification by ultracentrifugation for 2 h at 12 °C at 20,200 r.p.m. After ultracentrifugation, the pellet was resuspended in PBS for 16 h at 4 °C under constant agitation, aliquoted and frozen at –80 °C for later use.

Viral titration was performed by quantitative PCR (qPCR) with specific primers against the PBC-Psi region of the pLS-CG vector (listed in Supplementary Table 1). A standard curve was generated using serial dilutions of pLS-CG-d2EGFP-3′-UTR_tPA construct mixed with genomic DNA. The number of molecules was calculated using the formula: M=(*C*·6.02 × 10^23^)/(660·bp), where *C* is the concentration of the plasmid, bp the number of base pairs and *M* the number of vector molecules.

### Flow cytometry assay

Flow cytometry assays were performed in HPDE, RWP-1, PANC-1 and MIA PaCa-2 cells. Forty thousand cells were plated per well in 12-multiwell plates and, 24 h postseeding, cells were transduced with the indicated lentiviruses at 12 multiplicity of infection per virus. Transduction was facilitated by spinning for 2 h at 12,000 r.p.m. Two days post-transduction, d2EGFP and dRFP levels were analysed by flow cytometry using BD LSR II (Becton Dickinson). Flow cytometry results were analysed using FlowJo 8.7 for Macintosh.

### Adenovirus generation

The AdCPE genome was generated by the following steps: first, the E1A polyA sequence of the pEND-K plasmid was mutated from 5′-AATAAA-3′ to 5′-ACTCGA-3′, generating a new *Xho*I restriction site with the primer 5′-GCTGAATGAGATTGATGTAAGTTTACTCGAGGGTGAGATAATGTTTAACTTGC-3′ using the QuickChange Multi Site-Directed Mutagenesis Kit (Stratagene, Wilmington, NC, USA). Second, the construct containing E1A and the 3′-UTR of cB1 with the three CPE sequences was generated directly by PCR, by amplifying Adwt E1A with the primers 5′-CCTTGGGTCCGGTTTCTATGCC-3′ and 5′-CGTCTCGAGGCTTTATTAAAACCAGTAAAACATTAAAAACACAATACACTATTTACAGAAGCACATGGTGCAACACTTATGGCCTGGGGCGTTTACAGC-3′. Third, the E1A from the pEND-K with the mutated polyA sequence was replaced by the PCR product, using *Age*I and *Xho*I restriction sites, to generate pEND-K-E1A-cB1. Finally, AdCPE was generated by homologous recombination of pEND-K-E1A-cB1 with the genome of the serotype 5 wild-type adenovirus in *E. coli* BJ5183 cells as described^40^.

Adwt was obtained from ATCC. AduPAR was described previously^40^. AdDUC genome was generated by incorporating the CPE containing 3′-UTR of E1A into the *Box*I and *Afl*II restriction sites of the pSH-DM-UPAR-E1A plasmid followed by recombination of the resulting plasmid with the Adwt genome in BJ5183 cells as described previously^40^.

Adwt, AdCPE, AduPAR and AdDUC were propagated in A549 cells and purified by cesium chloride banding. The concentration of viral particles (vp ml^−1^) was determined by means of optical density, and infectious particles (pfu ml^−1^) were determined by hexon immunostaining in HEK293 cells^41^. Both viruses presented equal vp per pfu ratio.

### Western blot analysis

Protein extracts were obtained with lysis buffer (50 mM Tris-HCl at pH 6.8, 2% SDS) containing 1% Complete Mini Protease Inhibitor (Roche Diagnostics, Basel, Switzerland). BCA Protein Assay Kit (Pierce-Thermo Fisher Scientific, Waltham, MA, USA) was used to determine the protein concentration, and total proteins (35 μg) were resolved by electrophoresis on 7.5% gels and transferred to nitrocellulose membranes by standard methods. Membranes were immunoblotted with rabbit anti-adenovirus-2/5 E1A polyclonal antibody (1:200; clone 13S-5; Santa Cruz Biotechnology, Dallas, TX, USA) or anti-CPEB4 antibody (1:200; Abcam, Cambridge, UK) or anti-CPEB1 (1:200; 13274-1-AP; ProteinTech, IL, USA) 1 h at room temperature. Blots were rinsed with TBS-T and incubated for 45 min at room temperature with horseradish peroxidase-conjugated goat anti-rabbit immunoglobulin G (DakoCytomation, Glostrup, Denmark). Antibody labelling was detected by the enhanced chemiluminescent method (Amersham Biosciences, Amersham, UK). Western blot expression data for E1A, CPEB1 and CPEB4 were normalized to glyceraldehyde 3-phosphate dehydrogenase. Uncropped scans of western blots presented in the main figures are provided in Supplementary Fig. 11.

### cDNA synthesis and real-time qPCR

RNA was obtained and isolated using RNeasy Mini Kit (Qiagen, Venlo, The Netherlands). A total of 1 μg was reverse transcribed using Moloney murine leukemia virus reverse transcriptase and random decamers (Ambion, Carlsbad, CA, USA). One microlitre of the reaction was used as a template for the qPCR amplification reaction (LightCycler 480SYBER Green I Master Mix; Roche Diagnostics) in a thermocycler (ViiA 7 Real-Time PCR System; Applied Biosystems), using the following set of primers: E1A Fw, 5′-CGGCCATTTCTTCGGTAATA-3′ and E1A Rev, 5′-CCTCCGGTGATAATGACAAG-3′; Hexon Fw, 5′- GTCTACTTCGTCTTCGTTGTC-3′ and Hexon Rev, 5′-TGGCTTCCACGTACTTTG-3′; and Fibre Fw, 5′-CTCCAACTGTGCCTTTTC-3′ and Fibre Rv, 5′-GGCTCACAGTGGTTACATT-3′. Quantitative expression data were normalized to Gdx Fw, 5′-GGCAGCTGATCTCCAAAGTCCTGG-3′ and Gdx Rev, 5′-AACGTTCGATGTCATCCAGTGTTA-3′. d2EGFP and dRFP were detected with the primers: d2EGFP Fw, 5′-CAACAGCCACAACGTCTATATCAT-3′ and d2EGFP Rv, 5′-ATGTTGTGGCGGATCTTGAAG-3′; and dRFP Fw, 5′-GCCCTTCGCCTGGGACAT-3′ and dRFP Rv, 5′-GGTGCTTCACGTACACCTTGGA-3′. Quantitative expression data were normalized using the primers ACTB Fw, 5′-CTGGAACGGTGAAGGTGACA-3′ and ACTB Rv, 5′-GGGAGAGGACTGGGCCATT-3′.

### RNA-immunoprecipitation and RT–qPCR

RPE cells were transfected at 80% confluence with 25 μg of pCMV-lucRenilla plasmid (cB1-3′-UTR WT or cB1-3′-UTR Mut)^27^ using Lipofectamine LTX and PLUS Reagent (Thermo Fisher Scientific, Waltham, MA, USA) for 48 h. After transfection, cells were crosslinked with 0.5% formaldehyde in free DMEM for 5 min at room temperature. Then, cells were lysed with RIPA buffer supplemented with EDTA-free protease inhibitor cocktail (Sigma-Aldrich, Saint Louis, MO, USA) and 200 U ml^−1^ RiboLock RNAse Inhibitor (Thermo Fisher Scientific). Cell lysates were sonicated for 5 min at low intensity and centrifuged for 30 min at 13,200 r.p.m. and at 4 °C. Eight hundred micrograms of lysates were precleared with 20 ml of Dynabeads protein A (Thermo Fisher Scientific) for 30 min at 4 °C. Finally, they were incubated with 5 mg of anti-CPEB4 antibody (Abcam; ab83009) or anti-HuR antibody (Santa Cruz Biotechnology, sc-5261) or anti-immunoglobulin G antibody (Sigma-Aldrich) coupled to 50 μl of Dynabeads protein A overnight at 4 °C. One-fourth of the immunoprecipitates were eluted with Laemmli sample buffer by heating at 65 °C for 20 min, resolved in SDS–polyacrylamide gel electrophoresis and analysed by western blotting. Three-fourth of remaining immunoprecipitates were digested with proteinase K during 1 h at 65 °C, and the RNA was isolated by phenol extraction. All the RNAs were treated with DNAse (Thermo Fisher Scientific) and retrotranscribed with random hexamers and Oligo d(T)20, using SuperScript IV Reverse Transcriptase (Thermo Fisher Scientific) and subjected to qPCR. The resulting cDNAs were used for gene-specific qPCR, using the following primers: pLucORF Fw, 5′-ACTGGGACGAAGACGAACAC-3′; pLucORF Rv, 5′-GGCGACGTAATCCACGATCT-3′; Renilla S, 5′-GATAACTGGTCCGCAGTGGT-3′; Renilla AS, 5′-ACCAGATTTGCCTGATTTGC-3′. Fold enrichment of luciferase mRNA in the immunoprecipitated fraction was calculated after normalization with the gene expression from the inputs.

### Viral genome quantification

Viral DNA was obtained from supernatants, cellular extracts or frozen tissues using the UltraClean BloodSpin DNA Isolation Kit (Mo Bio Laboratories, Carlsbad, CA, USA) according to the manufacturer's instructions. Viral genomes were determined by real-time qPCR using the SYBER Green I Master plus mix (Roche Diagnostics) and the primers Hexon Fw, 5′-GCCGCAGTGGTCTTACATGCACATC-3′ and Hexon Rv, 5′-CAGCACGCCGCGGATGTCAAAG-3′. Adenoviral copy number was relativized to the cellular DNA content using the albumin intron 12 primers of Fw, 5′-CTGTCATCTCTTGTGGGCTGT-3′ and Rv, 5′-GGCTATCCAAACTCATGGGAG-3′.

### *In vitro* cell survival studies

Dose–response curves were constructed for all assessed cells transduced with doses ranging from 0.001 vp per cell to 10,000 vp per cell of Adwt or AdCPE. Cell viability was measured 3 days postinfection by a colorimetric assay following the manufacturer's instructions (MTT Ultrapure; USB, Cleveland, OH, USA).

### Polyadenylation assays

Polyadenylation patterns were evaluated using a modified version of the RNA ligation-coupled RT–PCR presented previously^42^. One microgram of total RNA was ligated to 0.1 μg of P1 anchor primer (5′-P-GGTCACCTTGATCTCAAGC-NH2-3′) in 10 μl reaction using T4 RNA ligase I (New England Biolabs, Ipswich, MA, USA) according to the manufacturer's instructions. Half of the reaction product was used in a 50 μl reverse transcription reaction with PrimeScript RT–PCR (Takara Bio, Mountain View, CA, USA) according to the manufacturer's instructions, using 0.1 μg of P1′ as a reverse primer (5′-GCTTCAGATCAAGGTGACCTTTTT-3′). An aliquot (2.5 μl) of this cDNA preparation was used for a first pre-PCR step with Fw-E1A-polyA primer (5′-GGTGTAAACCTGTGATTGCG-3′). One microlitre of this reaction was later used in each 25 μl PCR with the primers Fw-E1A-polyA and P1′.

### Toxicity analysis

PBS or 2 × 10^10^ vp of Adwt or AdCPE were injected intravenously into the tail vein of 6- to 8-week-old male immunocompetent C57BL/6 mice. Animals were weighed and examined daily for any clinical signs of toxicity. Three days later, mice were killed, organs were isolated and blood samples were collected by intracardiac puncture. Serum aspartate aminotransferase and alanine aminotransferase were determined on an Olympus AU400 Analyser (Olympus, Tokyo, Japan) at the Clinical Biochemistry Service, School of Veterinary Medicine, Autonomous University of Barcelona. All animal procedures met the guidelines of European Community Directive 86/609/EEC and were approved by the ethical committee (CEEA-University of Barcelona) and by the local authorities of the Generalitat de Catalunya.

### Mouse xenografts

RWP-1, MIA PaCa-2 and PANC-1 cells (2.5 × 10^6^), embedded in Matrigel 1:1 (BD Biosciences, San Jose, CA, USA), were subcutaneously injected into each flank of male, 7- to 8-week-old, athymic nu/nu mice (Harlan, Sant Feliu de Codines, Spain). Tumours were measured at least three times weekly, and their volumes were calculated using the formula *V*=larger diameter × (smaller diameter)^2^ × pi÷6. Mice were randomly assigned to either group for treatment. Virus was administered once tumours achieved a median volume of 100 mm^3^. The experimenter was blinded until the conclusion of the study.

### Statistical analyses

The descriptive statistical analysis was performed on GraphPad Prism v5.0a (GraphPad Software, La Jolla, CA, USA). Statistical differences were evaluated using nonparametric two-tailed Mann–Whitney test. *P*<0.05 was taken as the level of significance.

Statistical analyses for the d2EGFP/dRFP quantification were performed using R (version 2.10.0) and the ARM package, to perform the mixed model by REML and multcomp package for multiple comparison analysis of means by the Tukey–Kramer test.

Sample size calculation for animal studies took into consideration to have more than five mice in each group.

The *in vivo* tumour growth statistical analysis was evaluated using R v2.14.1 software (R: a language and environment for statistical computing; R Foundation for Statistical Computing, Vienna, Austria) with a linear mixed-effect model using the lme4 package. We associated a random-effects term with the day of measurement^43^. Statistical differences were evaluated using a multiple comparison of means by Tukey's contrasts.

### Data availability

All relevant data are available from the authors on reasonable request.

## Additional information

**How to cite this article:** Villanueva, E *et al*. Translational reprogramming in tumour cells can generate oncoselectivity in viral therapies. *Nat. Commun.* **8**, 14833 doi: 10.1038/ncomms14833 (2017).

**Publisher's note:** Springer Nature remains neutral with regard to jurisdictional claims in published maps and institutional affiliations.

## Supplementary Material

Supplementary Information Supplementary Figures and Supplementary Table
