# Supplementary material for: Translational reprogramming in tumour cells can generate oncoselectivity in viral therapies
Source: Nat Commun. 2017 Mar 16;8:14833. doi: 10.1038/ncomms14833 (PMC5357308; doi:10.1038/ncomms14833)
Supplement: Supplementary Information — Supplementary Figures and Supplementary Table [file ncomms14833-s1.pdf]

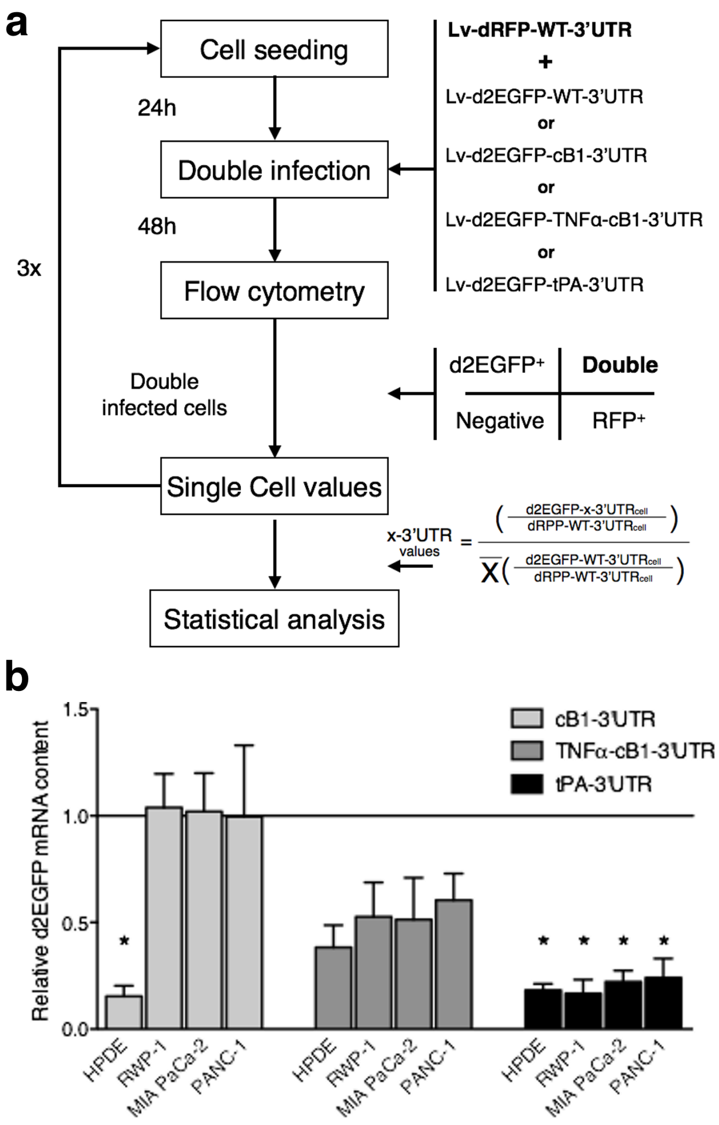

Supplementary Figure 1. (**a**) Experimental workflow of the d2EGFP/dRFP expression analysis from the different 3'UTRs in HPDE, RWP-1, PANC-1 and MIA PaCa-2 cells. (**b**) cB1 3'UTR reduces d2EGFP mRNA in HPDE cells but not in tumoral cells. Quantification of relative d2EGFP/dRFP mRNA levels in the indicated cell lines transduced with the indicated lentiviruses and relative to the mRNA content of d2EGFP/dRFP from Lv-WT 3'UTR transduced cells. Data is shown as mean ± SEM of three independent biological replicates, * $p<0.05$ (2-tailed Mann-Whitney test).

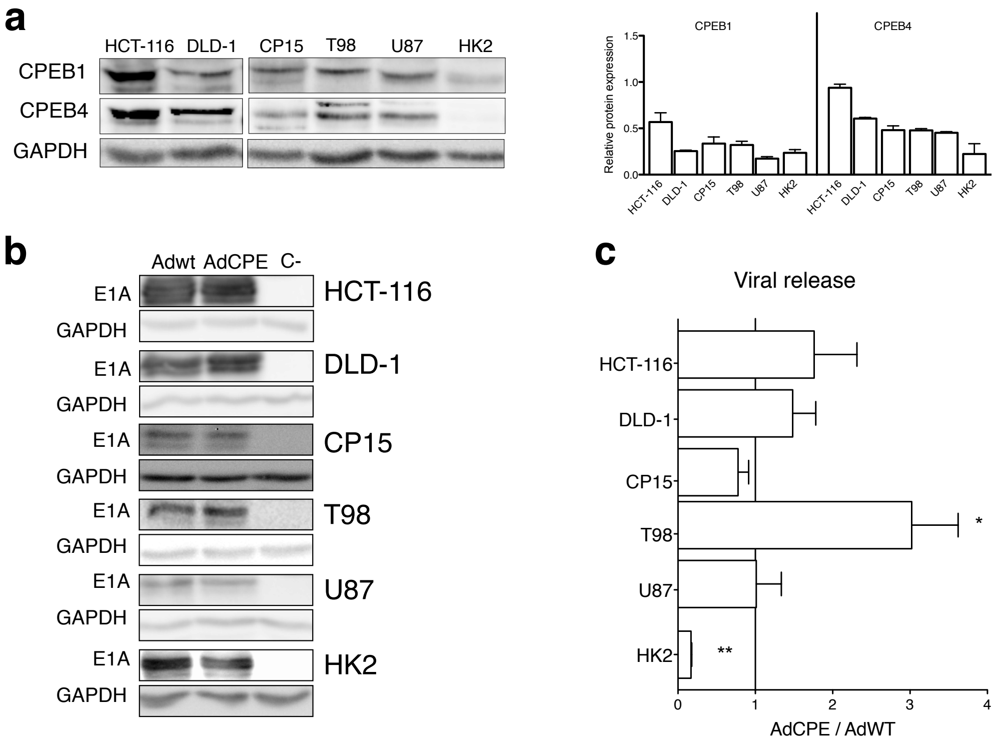

Supplementary Figure 2. AdCPE *In vitro* oncoselectivity. (**a**) (Left panel) Representative western blots showing CPEB1 and CPEB4 expression in cells from colorectal cancer (HCT-116, DLD-1), pancreatic cancer (CP15), glioblastoma (T98, U87) and the non tumoral human epithelial kidney cell line (HK2). (Right panel) Quantification of CPEB1 and CPEB4 signal normalized to GAPDH. (**b**) Representative E1A western blots of the indicated cell lines, infected with Adwt and AdCPE at 72 h PI. (c) Quantification of viral production in cell supernatants 72 h PI with Adwt and AdCPE, of tumoral (HCT-116, DLD-1, CP15, T98, U87) and non-tumoral (HK2) cells. qPCR data is shown as mean ± SEM of five independent biological replicates *p<0.05 and ** p<0.01 (2-tailed Mann-Whitney test).

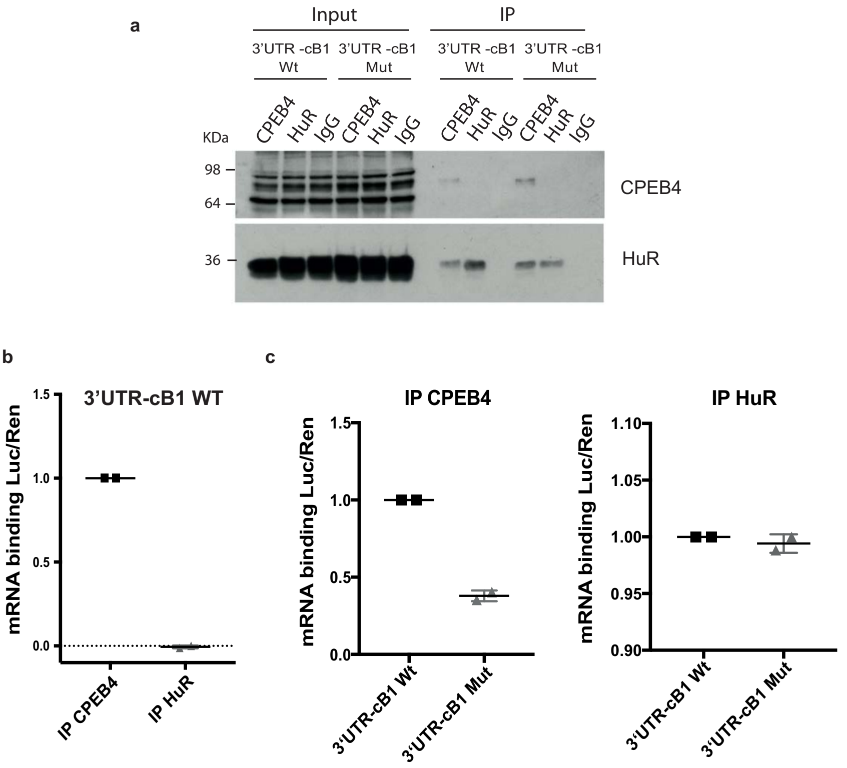

Supplementary Figure 3. RNA Immunoprecipitation (RIP) of CPEB4 and HuR in RPE cells transfected with 3'UTR cyclin B1 wild-type (3'UTR-cB1 Wt) or 3'UTR cyclin B1 CPE mutated (3'UTR-cB1 Mut). (**a**) Western Blotting analysis of CPEB4 and HuR shows the immunoprecipitation efficiency. IgG was used as negative control. (**b**) 3'UTR B1 WT mRNA levels were measured in CPEB4 and HuR immunoprecipitation by RT-qPCR. (**c**) The mRNA binding of 3'UTR-cB1 Wt and 3'UTR-cB1 Mut to CPEB4 or HuR was assessed by RT-qPCR. Results (in B and C) are shown as ratio Luciferase/Renilla (Luc/Ren) of Wt or Mut mRNA bound by CPEB4 or HuR. Data representative from three independent biological replicates.

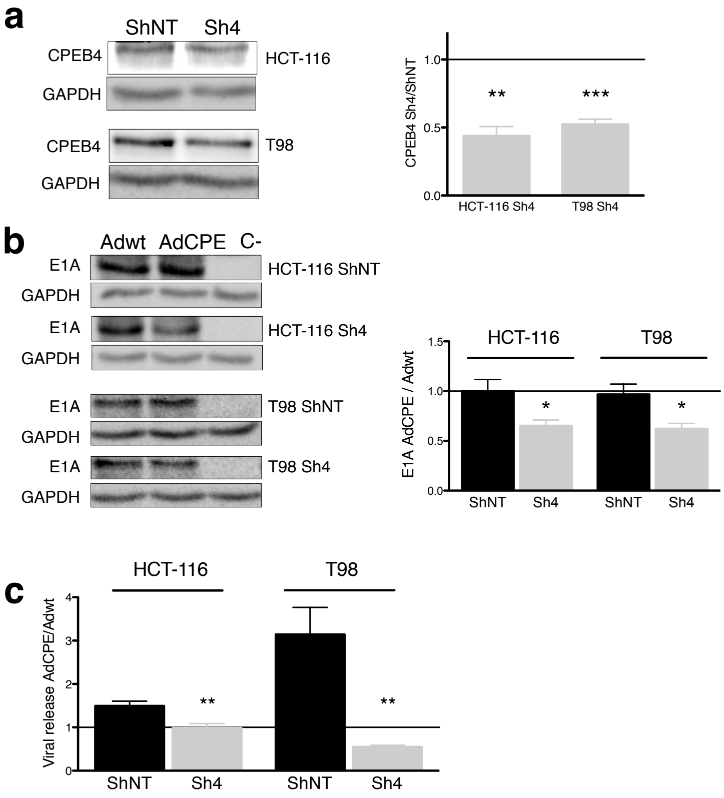

Supplementary Figure 4. CPEB4 regulates AdCPE E1A expression and viral fitness in colorectal cancer and glioblastoma cells lines. (**a**) Representative western blots of CPEB4 protein downregulation in HCT-116 and T98 tumoral cells. CPEB4 signal quantification normalized to GAPDH and expressed as relative values of CPEB4 Sh4/ShNT. (n=5). **p<0.01 and ***p<0.001. (**b**) Representative western blot of E1A expression in HCT-116 shNT, HCT-116 sh4, T98 ShNT and T98 Sh4 cells, infected with Adwt and AdCPE at 72 h PI. Quantification of E1A signal has been normalized to GAPDH and expressed as relative values of AdCPE/Adwt (n=5). *p<0.05. (**c**) Quantification of relative viral release (AdCPE / Adwt) in the supernatant of shNT and sh4 HCT-116 and T98 infected cells at 72 h PI. Data is shown as mean ± SEM of five independent biological replicates. ** p<0.01 (2-tailed Mann-Whitney test).

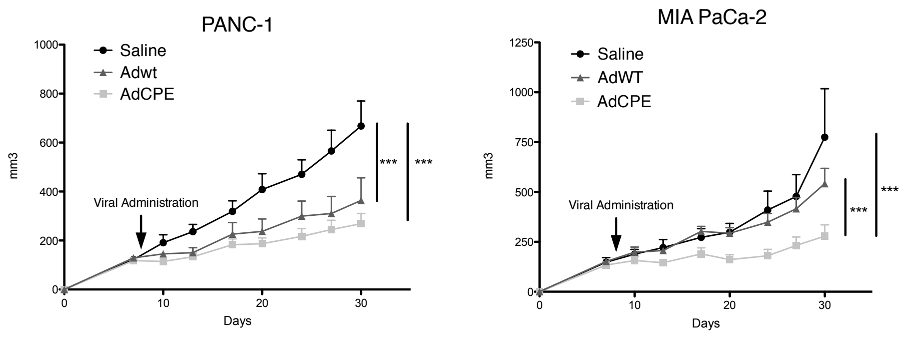

Supplementary Figure 5. AdCPE antitumor activity is similar or slightly superior than that of Adwt.

Follow-up of tumor volumes in mice bearing MIA PaCa-2 or PANC-1 xenografts intravenously treated with saline (n=8) or with a single injection of 2 x 10^{10}vp/mouse Adwt (n=8) or of 2x 10^{10}vp/mouse AdCPE (n=8). *** p<0.001 (Tukey contrast test on the lineal mixed model fitted by REML).

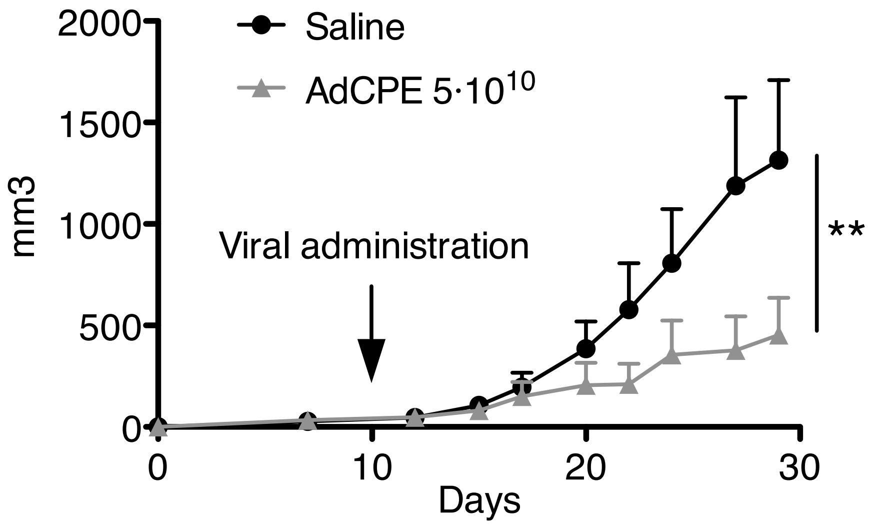

Supplementary Figure 6. Follow-up of tumor growth in mice bearing RWP-1 xenografts intravenously treated with saline (n=8) or a single injection of $5 \cdot 10^{10}$vp/mouse AdCPE (n=8). ** p<0.01 (Tukey contrast test on the lineal mixed model fitted by REML).

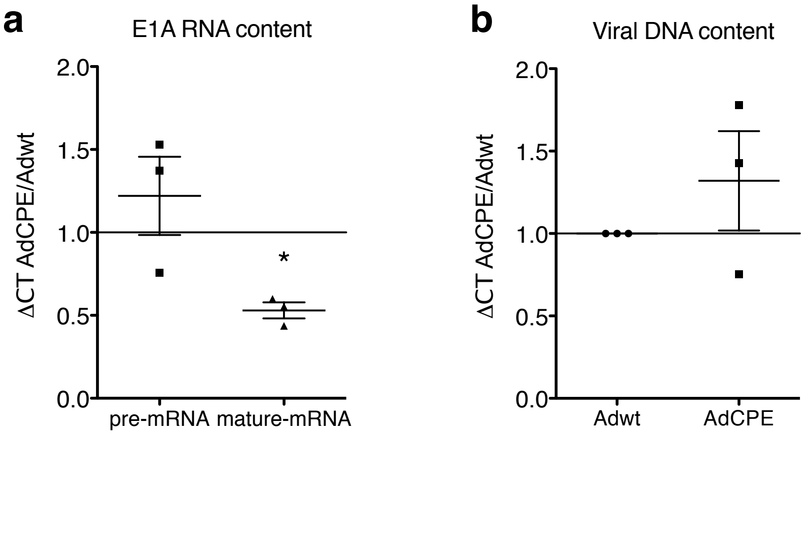

Supplementary Figure 7. AdCPE and Adwt E1A mRNA and viral particles quantification in mice livers. (**a**) Quantification of E1A pre-mRNA and mature mRNA in mice livers 4h post- viral administration. qPCR data is shown as mean ± SEM of three independent biological replicates * p<0.05 (2-tailed Mann-Whitney test). (**b**) Viral DNA quantification by qPCR in mice livers 4h post-viral administration.

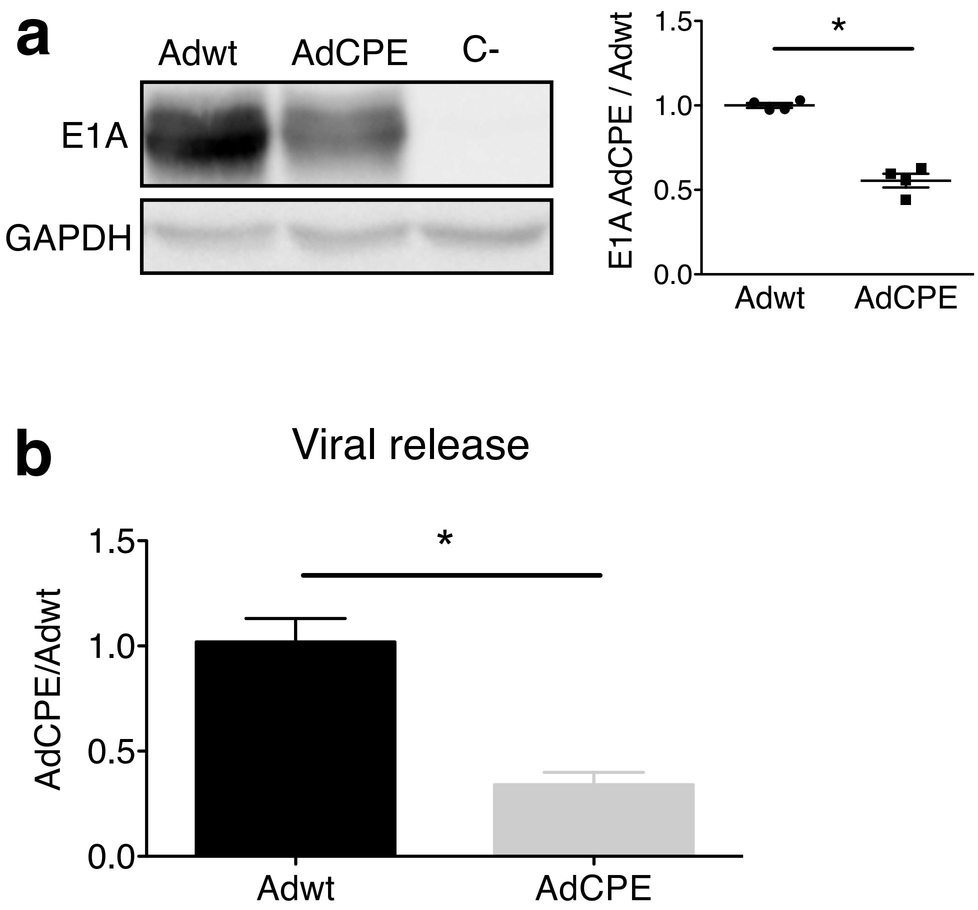

Supplementary Figure 8. AdCPE replication is attenuated in primary human hepatocytes. (**a**) Representative western blot of human hepatocytes infected with Adwt or AdCPE at 72 h PI. E1A signal has been normalized to GAPDH and expressed relative to Adwt values. (n=5). *p<0.05. (**b**) Quantification of relative viral production (AdCPE / Adwt) in the supernatant of human hepatocytes at 72 h PI. Data is shown as mean ± SEM of five independent biological replicates. * p<0.01 (2-tailed Mann-Whitney test).

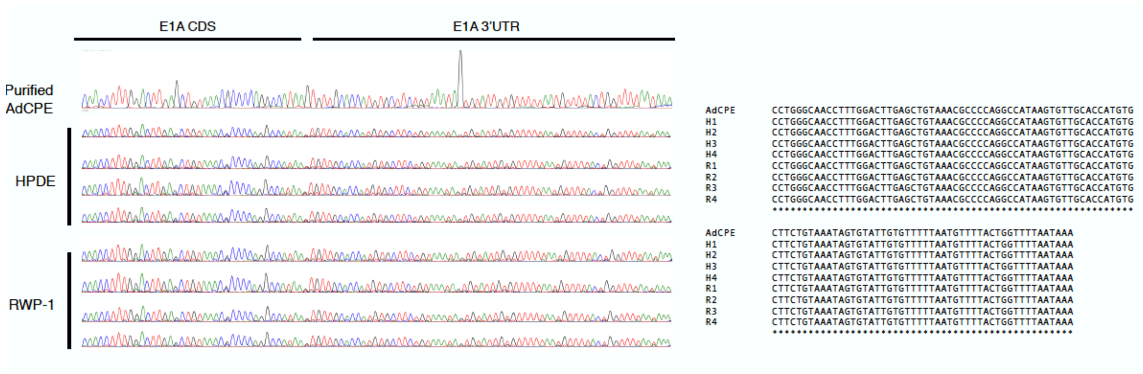

Supplementary Figure 9. CPE elements in the E1A 3’UTR of AdCPE remain stable after 20 consecutive viral replicative cycles. Sanger sequencing representation and sequence alignment of the E1A 3’UTR of AdCPE, between the purified virus and AdCPE isolated after 20 consecutive replicative cycles in HPDE (H) and RWP-1 (R) cells.

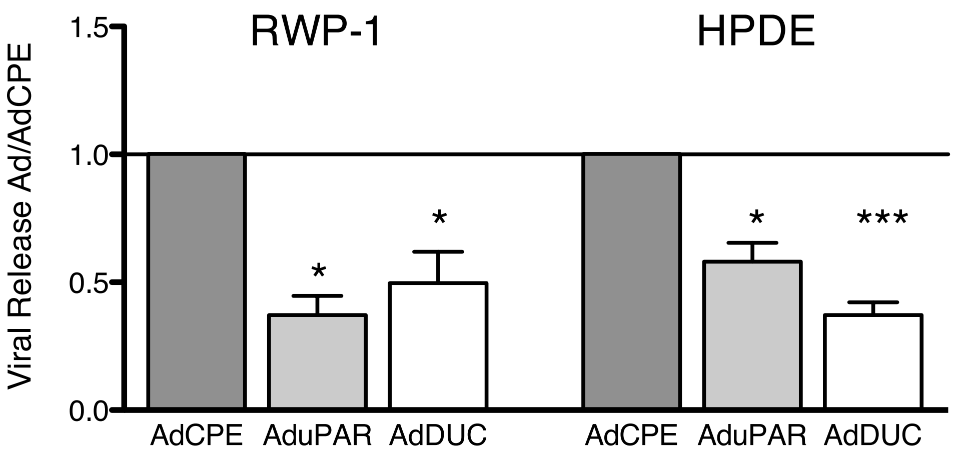

Supplementary Figure 10. AdDUC dual E1A-regulated virus with the uPAR promoter and the CPE elements display an oncoselective additive effect. qPCR quantification of viral particles in the supernatant of tumoral RWP-1 and non-tumoral HPDE cells at 72 h PI. Data is shown as mean ± SEM of five independent biological replicates. ** $p<0.01$ (one sample t-test).

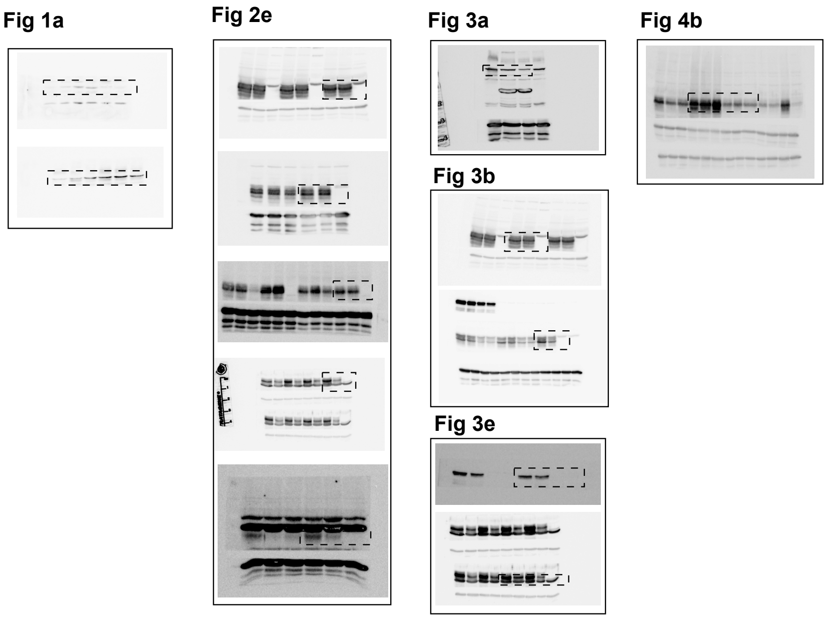

Supplementary Figure 11. Uncropped images of western blot figures shown in the main paper.

Supplementary Table 1: Primer sequences

d2EGFP	Fw	GCATACCGGTCGCCACCATGGTGAGCAAGGGCGAG
	RV	GGACTCGAGCTACACATTGATCCTAGCAG
dRFP	Fw	CGGGGATCCACCGGTCGCCACCATG
	RV	CTCGAGCCGCCTACACATTGATCCTAGC
CB1	Fw	GCATACCGGTCGCCACCATGGTGAGCAAGGGCGAG
	RV	CGTCTCGAGGCTTTATTAAAACCAGTAAAACATTAAAAACACAATACACTATTTACAGAAGCACATGGTGCAACACTACACATTGATCCTAGCAG
tPA	Fw	CTCGAGTATATTTATAGTCACAAGAGC
	RV	CTCGAGGAAAAACTGTGAAAAATATAT
ARE	Fw	GCTACTCTCGAGAGCTGAGTTAACATTATTTATTATTTATTTATTATTTATTTATTTATGTTGCACCATGTGCTTCTG
	Rv	GATCCTACTAGTAGGATCTCGA
pLS-CG- PBS-Psi	Fw	ACCTGAAAGCGAAAGGGAAAC
	Rv	CACCCATCTCCTCCTTCTAGCC
PLS-CG mut_4193-4199		GAAGCACTCAAGGCAAGCTTGGTTGAGGCTTAAGCAGTGGG
pLS-CG MUT_4423-4429		GCGATGCAATTTCCTCATTTGGTTAGGAAAGGACAGTGGG
